# Clinically Relevant Transmitted Drug Resistance to First Line Antiretroviral Drugs and Implications for Recommendations

**DOI:** 10.1371/journal.pone.0090710

**Published:** 2014-03-17

**Authors:** Susana Monge, Vicente Guillot, Marta Alvarez, Natalia Chueca, Natalia Stella, Alejandro Peña, Rafael Delgado, Juan Córdoba, Antonio Aguilera, Carmen Vidal, Federico García, CoRIS

**Affiliations:** 1 National Centre of Epidemiology, Madrid, Spain; 2 Microbiology Department, San Cecilio University Hospital, Instituto Biosanitario de Investigación (IBIG), Granada, Spain; 3 Infectious Diseases Unit, La Paz University Hospital/IdiPAZ, Madrid, Spain; 4 Microbiology Department, Doce de Octubre University Hospital, Madrid, Spain; 5 Microbiology Department, La Fe University Hospital, Valencia, Spain; 6 Microbiology Department, University Hospital of Santiago, Santiago de Compostela, Spain; 7 Microbiology Department, Son Espases University Hospital, Palma de Mallorca, Spain; Centro de Biología Molecular Severo Ochoa (CSIC-UAM), Spain

## Abstract

**Background:**

The aim was to analyse trends in clinically relevant resistance to first-line antiretroviral drugs in Spain, applying the Stanford algorithm, and to compare these results with reported Transmitted Drug Resistance (TDR) defined by the 2009 update of the WHO SDRM list.

**Methods:**

We analysed 2781 sequences from ARV naive patients of the CoRIS cohort (Spain) between 2007–2011. Using the Stanford algorithm “Low-level resistance”, “Intermediate resistance” and “High-level resistance” categories were considered as “Resistant”.

**Results:**

70% of the TDR found using the WHO list were relevant for first-line treatment according to the Stanford algorithm. A total of 188 patients showed clinically relevant resistance to first-line ARVs [6.8% (95%Confidence Interval: 5.8–7.7)], and 221 harbored TDR using the WHO list [7.9% (6.9–9.0)]. Differences were due to a lower prevalence in clinically relevant resistance for NRTIs [2.3% (1.8–2.9) vs. 3.6% (2.9–4.3) by the WHO list] and PIs [0.8% (0.4–1.1) vs. 1.7% (1.2–2.2)], while it was higher for NNRTIs [4.6% (3.8–5.3) vs. 3.7% (3.0–4.7)]. While TDR remained stable throughout the study period, clinically relevant resistance to first line drugs showed a significant trend to a decline (p = 0.02).

**Conclusions:**

Prevalence of clinically relevant resistance to first line ARVs in Spain is decreasing, and lower than the one expected looking at TDR using the WHO list. Resistance to first-line PIs falls below 1%, so the recommendation of screening for TDR in the protease gene should be questioned in our setting. Cost-effectiveness studies need to be carried out to inform evidence-based recommendations.

## Introduction

HIV drug resistance due to transmitted mutations in the reverse transcriptase (RT) and protease (Pro) regions has been associated with a higher risk of virological failure to first line antiretroviral therapy (ART) [1], having a greater impact for initial regimens containing a non-nucleoside reverse transcriptase inhibitor (NNRTI) [2]. Testing for transmitted drug resistance (TDR) in newly diagnosed patients with HIV is strongly recommended by treatment guidelines [3]–[6], as it has shown to be cost-effective, in terms of gain in quality-adjusted life year (QALYs) when drug resistance prevalence is over 1–5% [7].

The Spanish cohort of naïve HIV infected individuals (CoRIS) offers relevant information about the current epidemiological profile of HIV infection [8], [9], and is an excellent scenario to characterise the prevalence of TDR over time in Spain. Two previous analyses of viral sequences in CoRIS were carried out for the periods 2004–2008 and 2007–2010, and have been published elsewhere [10], [11]. For these two previous studies, we used the 2009 update World Health Organization (WHO) comprehensive list of mutations [12], which has been also widely used for TDR evaluation [13]–[16]

WHO mutations list has overcome the major limitation of TDR studies across the world, thus providing high levels of standardization into these studies. However, it defines TDR to the different classes of antiretroviral drugs based on the presence of at least one mutation related to drug resistance, while first line treatment drugs that are currently approved by many of the latest updates of clinical guidelines, often include compounds, for which more than one mutation is necessary in order to reduce in vivo drug activity (e.g, abacavir, and boosted Protease Inhibitors). Further approaches, such as the Stanford HIV Drug Resistance Database algorithm, calculate the effective resistance given the combination of mutations present in a particular strain, allowing analyzing clinically relevant resistance to specific antiretroviral drugs (ARVs) and regimens, providing a complementary and invaluable input for informing clinical recommendations.

The objective of this study was to analyse clinically relevant resistance to drugs included in recommended first-line regimens in Spain (CoRIS) from 2007 to 2011, using the Stanford algorithm, and compare it to TDR, defined by the WHO list of mutations.

## Patients and Methods

CoRIS is an open, multicentre, prospective cohort of HIV-positive, antiretroviral-naïve subjects over 13 years of age, including both seroprevalent and seroconverter patients. Subjects are recruited and followed up in 31 HIV units from 13 of the 17 Autonomous Communities of Spain. Ethics approval was obtained from participating sites and a written informed consent was obtained from every patient included in the study. Detailed descriptions of the cohort have been previously published [8], [9]. As part of the cohort data collection process, which began in 2004, sites are asked every year to provide a FASTA viral sequence, encoding the HIV protease and RT obtained at the time of inclusion, available from routine resistance testing. As of October 2011, 23 sites from 10 Autonomous Communities were collaborating in the collection of FASTA sequences. In the cohort-coordinating centre, these sequences are linked to clinical and epidemiological data of the patient. Further, the cohort coordinating centre double checks that dates of FASTA sample collection are previous to initiation of ART. Study period for this analysis was from January 2007 to October 2011 and only naïve patients with availability of a FASTA sequence of medium or high quality were included. Sequence data may be available to qualified researchers upon request.

Clinically relevant drug resistance to the first line antiretrovirals (ARVs) included in recommended first-line regimens in Spain as of current guidelines [6] [abacavir, emtricitabine, lamivudine and tenofovir, (Nucleoside Analogue Reverse Transcriptase Inhibitors, NRTIs), efavirenz and nevirapine (Non-Nucleoside Analogue Reverse Transcriptase Inhibitors, NNRTIs), atazanavir, darunavir and lopinavir (Protease Inhibitors, PIs)] was evaluated using the Stanford Database algorithm [17]. For analysis, Stanford HIVdb “Low-level resistance”, “Intermediate resistance” and “High-level resistance” categories were considered as “Resistant”, while “Susceptible” and “Potential Low Level Resistance” were pooled into Susceptible. For subsequent genotypic sensitivity score (GSS) analysis, “Susceptible” and “Potential Low Level Resistance” were scored as 1, “Low Level Resistance” and “Intermediate Resistance” were pooled into Intermediate and scored as 0.5, and “High Level Resistance” was scored as 0. GSS was calculated adding individual resistance scores for each first-line drug in the regimen.

Alternatively, transmitted drug resistance (TDR) associated mutations were evaluated following the WHO surveillance drug resistance mutation list updated in 2009 by Bennett and colleagues [12].

The sample was described using proportion or median (interquartile range) for categorical and continuous variables, respectively; and bivariate analysis was performed using Chi Square or Kruskall-Wallis test as appropriate. Resistance mutations were described using prevalence, and the corresponding confidence intervals were calculated with an analytically derived variance estimator. Their linear trend over the study period was analysed using chi-square test for trend. Significance level was 5%. All the analyses were conducted using Stata software (V.11.1, Stata Corporation, College Station, Texas, USA).

## Results

A total of 23 out of the 28 centres in CoRIS contributed FASTA sequences from 2007 to 2011. These centres provided 7,351 patients, 92.2% of the CoRIS cohort, and a complete naïve sequence was submitted for 2,781 (37.8%). Compared to the total cohort, those with an available FASTA sequence were younger (median age 33.9 vs. 34.9), more frequently males (88.3% vs. 83.9%), infected through sex between men (69.2% vs. 58.9%), with a higher educational level (63.5% vs. 56.2%), originating from Spain compared to other regions (70.1% vs. 68.0%), and recruited in earlier stages of HIV disease progression (CD4 count at recruitment 424 vs. 323 cells/mm^3^). All differences were statistically significant at p<0.01.

A description of the study population by year in which sample for sequencing was obtained is shown in Table 1. An increase in the proportion of men who have sex with men (MSM), of subjects with a higher education, and of patients at earlier CDC stages and higher CD4 counts was observed over time. Median time from cohort entry to resistance study was -1 day (IQR: -21 to 13).

**Table 1:** Baseline characteristics of the study population.

		2007		2008		2009		2010		2011		Total		
		(N = 484)		(N = 582)		(N = 596)		(N = 756)		(N = 363)		(N = 2,781)		p
		n	(%)	n	%	n	%	n	%	n	%	n	%	
Sex (Male)		436	90.1	481	82.6	529	88.8	683	90.3	326	89.8	2,455	88.3	<0.01
Age*, Median (IQR)		33.8 (28.3–41.0)		34.3(28.2–41.5)		33.2(28.0–39.7)		34.5(28.5–41.6)		33.8(27.3–42.3)		33.9 (28.2–41.0)		0.37
Mode of transmission	IDU	35	7.2	47	8.1	27	4.5	38	5.0	14	3.9	161	5.8	
	MSM	321	66.3	347	59.6	429	72.0	550	72.8	278	76.6	1,925	69.2	<0.01
	Heterosexual	119	24.6	178	30.6	126	21.1	140	18.5	63	17.4	626	22.5	
	Other/NA	9	1.9	10	1. 7	14	2.4	28	3.7	8	2.2	69	2.5	
Country of origin	Spain	331	68.4	408	70.1	435	73.0	513	67.9	262	72.2	1,949	70.1	
	Africa	22	4.5	23	4.0	27	4.5	23	3.0	7	1.9	102	3.7	0.07
	Latin America	106	21.9	108	18.6	106	17.8	174	23.0	67	18.5	561	20.2	
	Other/unknown	25	5.2	43	7.4	28	4.7	46	6.1	27	7.4	169	6.1	
Educational level	Lower	127	26.2	175	30.1	148	24.8	191	25.3	88	24.2	729	26.2	
	Higher	299	61.8	341	58.6	380	63.7	503	66.5	244	67.2	1,767	63.5	<0.05
	Unknown	58	12.0	66	11.3	68	11.4	62	8.2	31	8.5	285	10.3	
Viral Load* (log c/ml)	N	349		429		440		622		291		2,131		0.11
	Median (IQR)	4.5 (4.0–5.1)		4.6 (4.0–5.0)		4.6 (4.0–5.1)		4.6 (4.1–5.1)		4.7 (4.2–5.2)		4.6 (4.1–5.1)		
CD4 count * (cells/mm3)	N	416		501		503		699		330		2,449		<0.05
	Median (IQR)	392 (237–604)		371 (223– 559)		396 (251– 593		413 (276–580)		432(260–606)		399 (251–588)		
Duration of the infection*	Recent infection	43	8.9	47	8.1	51	8.6	70	9.3	34	9.4	245	8.8	
	Chronic infecion	112	23.1	121	20.8	138	23.1	169	22.3	76	20.9	616	22.2	0.96
	Not evaluable	329	68.0	414	71.1	407	68.3	517	68.4	253	69.7	1,920	69.0	
CDC stage*	P/A	402	83.6	492	84.8	537	90.2	658	87.2	326	89.8	2,415	87.1	
	B	40	8.3	38	6.6	29	4.9	53	7.0	23	6.3	183	6.6	<0.05
	C	39	8.1	50	8.6	29	4.9	44	5.8	14	3.9	176	6.3	
History of delayed diagnosis	Yes	149	30.8	205	35.2	169	28.4	218	28.8	114	31.4	855	30.7	
	No	248	51.2	277	47.6	325	54.5	439	58.1	192	53.0	1,481	53.3	<0.05
	Not evaluable	87	18.0	100	17.2	102	17.1	99	13.1	57	15.7	445	16.0	

*At the time of the resistance testing; IDU: injecting drug users; MSM: men who have sex with men; P/A: Primoinfection or CDC stage A; NA: Not available; IQR: interquartile range.

### Transmitted Drug Resistance (TDR) according to the WHO list

**Figure 1:**
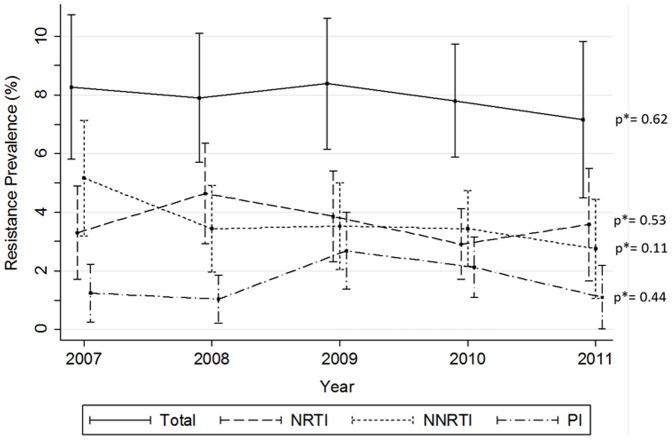
Prevalence of TDR (WHO SDRM estimates) by antiretroviral class between 2007 and 2011. NRTI: nucleoside reverse transcriptase inhibitors; NNRTI: non-nucleoside reverse transcriptase inhibitors; PI: protease inhibitors. * p value for Chi-square test for trend.

A total of 221 patients had at least one mutation among those contained in the WHO surveillance list, giving a prevalence of TDR of 7.9% (95%Confidence Interval: 6.9–9.0). Regarding mutations affecting specific ARV classes, similar prevalence of TDR was found for nucleoside reverse transcriptase inhibitors (NRTIs) and non-NRTIs (NNRTIs), being 3.6% (2.9–4.3) for NRTIs and 3.7% (3.0–4.7) for NNRTIs. TDR to protease inhibitors (PIs) was less prevalent, of 1.7% (1.2–2.2). Figure 1 shows prevalence of TDR for each ARV class throughout the study period. TDR to NNRTIs decreased from 5.2% in 2007 to 2.8% in 2011, although the trend was not statistically significant. TDR to more than one class of ARV was uncommon; 1.0% (0.6–1.3) and 0.1% (0.0–0.2) of the subjects showed TDR to a minimum of two or three ARV classes, respectively. TDR to two ARV classes decreased from 1.5% in 2007 to 0.3% in 2011, but again was not statistically significant.

Table 2 shows prevalence of specific mutations in the WHO surveillance list. The main mutation related to TDR to NNRTI was K103N/S, which had a prevalence of 2.8% (2.2–3.4). Three mutations were identified as being mainly responsible for TDR to NRTIs: T215 revertants (C/D/E/N/I/V/S) (1.3%; 0.9–1.8), M41L (1.2%; 0.8–1.5) and K219EQNR (1.0%; 0.7–1.4). For PIs, the mutation mainly responsible for TDR was M46IL (0.9%; 0.6–1.3). Of interest, a single mutation (singleton) was responsible for TDR to NRTIs, NNRTIs or PIs in 55.5%, 85.3% and 93.8% of the cases.

**Table 2:** Prevalence of mutations from the WHO transmitted drug resistance surveillance list.

NRTI mutations			NNRTI mutations			PI mutations				
Mutation	N	(Pv, %)	Mutation	n	(Pv, %)	Mutation		n		(Pv, %)
M41L	32	(1.15)	L100I	2	(0.07)	L24I	2		(0.07)	
K65R	1	(0.04)	K101EP	8	(0.29)	D30N	1		(0.04)	
D67EGN	23	(0.83)	K103N/S	77	(2.77)	V32I	2		(0.07)	
T69D	5	(0.18)	Y181CIV	9	(0.32)	M46IL	26		(0.93)	
K70ER	5	(0.18)	Y188CHL	5	(0.18)	I47AV	2		(0.07)	
L74IV	3	(0.11)	G190AES	11	(0.40)	F53LY	2		(0.07)	
F77L	2	(0.07)	P225H	3	(0.11)	I54ALMSTV	2		(0.07)	
Y115F	2	(0.07)	M230L	1	(0.04)	V82ACFLMST	5		(0.18)	
M184IV	13	(0.47)				N83D	1		(0.04)	
L210W	12	(0.43)				I85V	1		(0.04)	
T215REV*	37	(1.33)				N88DS	2		(0.07)	
T215YF	2	(0.07)				L90M	10		(0.36)	
K219EQNR	29	(1.04)								
1 mut.	56	(2.01)	1 mut.	87	(3.13)	1 mut.	45		(1.62)	
2 mut.	32	(1.15)	2 mut.	13	(0.47)	2 mut.	2		(0.07)	
≥3 mut.	13	(0.47)	≥3 mut.	2	(0.07)	≥3 mut.	1		(0.04)	
Prevalence (95%CI)	3.6	(2.9–4.3)	Prevalence (95%CI)	3.7	(3.0–4.4)	Prevalence (95%CI)	1.7		(1.2–2.2)	

NRTI: nucleoside reverse transcriptase inhibitors; NNRTI: non-nucleoside reverse transcriptase inhibitors; PI: protease inhibitors; Pv: prevalence; mut.:mutation. Amino acide abbreviations: A, alanine; C, cysteine; D, aspartate; E, glutamate; F, phenylalanine; G, glycine; H, histidine; I, isoleucine; K, lysine; L, leucine; M methionine; N, asparagine; P, proline; Q, glutamine; R, arginine; S, serine; T, threonine; V, valine; W, tryptophan; Y, tyrosine. * = C/D/E/N/I/V/S.

### Clinically relevant resistance to first-line drugs according to the Stanford Algorithm

When investigating clinically relevant resistance to ARV drugs that make part of recommended initial regimens, the number of patients that showed any resistance was 188, for a total prevalence of 6.8% (5.8–7.7). Prevalence of clinically relevant resistance to any first line ARV was lower than TDR for NRTIs [2.3% (1.8–2.9)] and PIs [0.8% (0.4–1.1)], while it increased in the case of NNRTIs to reach 4.6% (3.8–5.3). In fact, only 70.1% of patients harboring TDR actually showed any clinically relevant resistance to first-line drugs. Percentages for NRTIs and PIs were 65.3% and 35.4%, respectively. For NRTIs, the mutations responsible for having TDR, but without clinically relevant resistance to first line drugs, were present mainly as singletons: a Thymidine Associated Mutation (TAM) alone (M41L, n = 8; T69D, n = 1; K70R, n = 1; L210W, n = 4; K219N/Q, n = 7), a T215 revertant alone (D/N/S n = 7), a F77L alone (n = 2), or a combination of D67N+K219Q (n = 4), or D67N+T69D (n = 1). For PIs, mutations responsible for these discordances were always detected as singletons (L24I, n = 1; D30N, n = 1; M46I/L, n = 24; F53Y, n = 1, V82L, n = 2 and N88D, n = 2). On the other hand, the increase in clinically relevant resistance for NNRTIs was driven mainly by the combination of two or more mutations that are not present in the WHO list (V90I+V108I+H221Y, n = 1; A98G+V179E, n = 1; V108I+E138A, n = 3; V108I+V179E, n = 2; E138A+V179D, n = 2; E138A+V179D+Y188H, n = 1; V179D+ V106I, n = 1), and in 7 cases by the presence of a A98G singleton.

**Figure 2:**
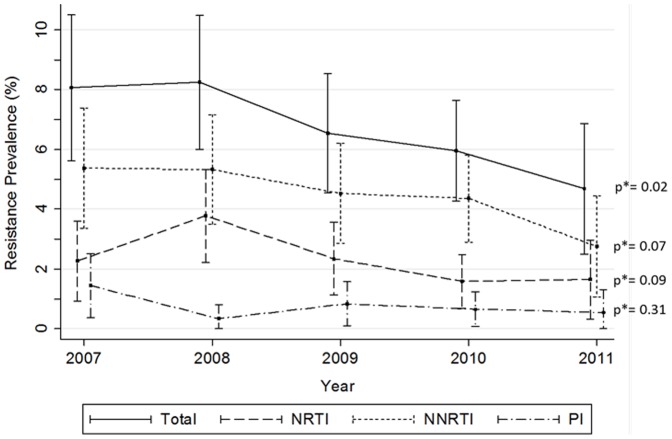
Prevalence of clinically relevant resistance to first line drugs (Stanford HIVdb) by antiretroviral class between 2007 and 2011. NRTI: nucleoside reverse transcriptase inhibitors (abacavir, emtricitabine, lamivudine and tenofovir); NNRTI: non-nucleoside reverse transcriptase inhibitors (efavirenz and nevirapine); PI: protease inhibitors (atazanavir, darunavir and lopinavir). * p value for Chi-square test for trend.

Figure 2 shows the prevalence of clinically relevant resistance to first line ARV drugs by ARV class. A significant decrease (p = 0.02) was found from 2007 (8.1%) to 2011 (4.7%), explained by a borderline statistical significance decrease in both NRTIs and NNRTI. No trend was found in case of PIs. Primary drug resistance to two or three ARV classes was low, of 0.8% (0.5–1.2) and of 0.04% (0.0–0.1), respectively.

For individual first line NRTIs, clinically relevant resistance was 2.2% (1.7–2.8) for abacavir, 0.7% (0.4–1.0) for emtricitabine and lamivudine, and 1.6% (1.1–2.1) for tenofovir; for first line NNRTIs, resistance was observed in 4.0% (3.3–4.8) for efavirenz and 4.6% (3.8–5.3) for nevirapine; finally, for PIs, resistance was observed in 0.7% (0.4–1.0) for atazanavir, 0.1% (0.0–0.3) for darunavir, and 0.3% (0.1–0.5) for lopinavir.

### Therapeutic barrier to resistance of first-line drug regimens

Table 3 shows resistance when combining these drugs into first line recommended regimens. Overall, 188 patients (6.8%) showed resistance to at least one regimen (GSS ≤3). Most affected regimens were those containing NNRTIs with patients showing resistance between 5.6% and 6.2%, depending on the specific combination of drugs. Resistance to PI based regimens was less common, between 2.2% and 2.7%. Of note, only 0.7% to 0.9% of the PI containing regimens showed two or less fully active drugs.

**Table 3:** Therapeutic barrier of first line ARV regimens using Stanford Algorithm.

ARV regimen			Genotypic Sensitivity Score			
			<3		≤2	
			n	(%)	n	(%)
EFV	TDF	FTC/3TC	155	(5.6)	94	(3.4)
	ABC	FTC/3TC	157	(5.7)	95	(3.4)
NVP	TDF	FTC/3TC	169	(6.1)	109	(3.9)
	ABC	FTC/3TC	171	(6.2)	109	(3.9)
LPV	TDF	FTC/3TC	64	(2.3)	20	(0.7)
	ABC	FTC/3TC	68	(2.5)	20	(0.7)
ATZ	TDF	FTC/3TC	72	(2.6)	25	(0.9)
	ABC	FTC/3TC	75	(2.7)	26	(0.9)
DRV	TDF	FTC/3TC	61	(2.2)	19	(0.7)
	ABC	FTC/3TC	65	(2.3)	19	(0.7)

* Threshold of the genotypic sensitivity score (GSS) used to consider the strain as resistant to a given ARV combination. TDF: tenofovir; ABC: abacavir; 3TC: lamivudine; FTC: emtricitabine; EFV: efavirenz; NVP: nevirapine; LPV: lopinavir; DRV: darunavir; ATZ: atazanavir.

## Discussion

Our study shows that, in Spain for the period 2007–2011, the prevalence of clinically relevant resistance to approved first line ARV drugs analyzed using the Stanford HIV interpretation system is lower than the one we would expect when looking at the Transmitted Drug Resistance (TDR) defined in the WHO mutations list. Moreover, a significant trend to a reduction in resistance was found, driven by a decrease in resistance to the first line NNRTIs efavirenz and nevirapine, as well as to the NRTIs abacavir, emtricitabine, lamivudine, and tenofovir, while resistance to the PIs atazanavir, darunavir and lopinavir, remained stable and very low throughout the whole period. In contrast, no significant trend in the TDR defined by WHO list has been observed. Resistance to more than one antiretroviral class was very uncommon by year 2011.

Many studies have evaluated trends in TDR transmission across Europe and the US [10], [11], [13]–[16], [18]–[21]. The use of the 2009 update of the WHO surveillance recommendations for the estimation of TDR has brought uniformity to all TDR surveys, and has had the great benefit of making all these studies comparable. It also has proved extremely useful for characterizing the epidemiology of TDR and carrying out population-based surveillance. However, its correlation with resistance to the different ARVs is not always straightforward, and further analysis of these mutations such as the one proposed by the Stanford HIV Resistance Database, can provide a complementary and useful input to clinicians, public health professionals and policy makers to develop evidence-based clinical guidelines.

In our study, TDR was of 7.9%, higher than clinically relevant resistance to first line drugs (prevalence of 6.8%). This shows that effective resistance to first line regimens in Spain would lie below the one we would expect when looking at the list of resistance mutations surveillance. In fact, up to 30% of patients harboring TDR did not actually show clinically relevant resistance, being this percentage higher in the case of PIs (65%) and NRTIs (34%). Specifically looking at clinical resistance to first line drugs is therefore relevant and can have important implications.

Clinically relevant resistance to first line PIs remained below 1% for all years except 2007, and was mostly of intermediate level, showing that transmitted resistance to PIs is not threatening effectiveness of PI-based first line ART in Spain. In agreement with what was previously suggested by an Irish report [14], our study greatly supports the hypothesis that there is no need from the clinical point of view to screen for PI resistance in our setting, though having the protease sequence may be useful for epidemiologic studies tracing TDR. A recent study has reported a misinterpretation of resistance to PIs, with an increase in the level of transmitted resistance to this class of antiretrovirals when using the WHO list [22]. In our study we have found that focusing only on TDR may also overestimate the impact of circulating resistances on the clinical effectiveness of first-line PI-containing regimens. In fact, 93.8% of the patients with TDR to PIs harboured a single mutation (singleton) in the protease. For classes including drugs with a high genetic barrier to resistance, such as PIs [23], the presence of just one mutation should not preclude its further use for first line regimens. Specific studies on clinically relevant mutations would be needed for informing this decision.

In a similar way, we have also shown this effect for NRTIs, with a prevalence of TDR of 3.6%, and a prevalence of clinically relevant resistance to first line drugs of 2.3%. Again, singletons were responsible for these differences (55.5% of the patients with TDR to NRTIs harboured a single mutation). Abacavir and tenofovir are the two drugs in the NRTI class with the best resistance profile, being the least affected drugs by single mutations. In fact most of the discordant cases showing TDR showed resistance to zidovudine or stavudine, two NRTIs that are no longer included in Spanish and most international recommendations for first line treatment. In contrast, the NNRTI class, represented for first line with two low genetic barrier drugs, showed higher clinically relevant resistance than TDR. This difference was mainly driven by intermediate resistance calls for efavirenz and/or nevirapine by a combination of two or more mutations that are not included in the WHO list, or by the presence of A98G alone.

Evaluating resistance to specific drugs also allows evaluating the global therapeutic barrier of a regimen, measuring the global robustness to resistance of a certain combination rather than resistance to a certain drug. This may be of extreme importance for evaluating clinical resistance to first line regimens, establishing recommendations on how resistance testing should be performed on newly diagnosed patients, and updating treatment guidelines. In our study, first line NNRTI containing regimens showed the lowest therapeutic barrier, as 5.6% to 6.2% of the newly diagnosed patients from CoRIS, showed any degree of resistance to at least one of the drugs in the regimen. In contrast, the therapeutic barrier to first line PI-based regimens was much higher, with between 2.2% and 2.7% showing any degree of resistance, and only 0.7% to 0.9% of the patients with two or less active drugs in the regimen, most of them being fully resistant to emtricitabine or lamivudine. To our knowledge, this is the first study to estimate the therapeutic barrier of first line regimens, and our results reinforce the previously mentioned notion that, in our setting, a reverse transcriptase-only approach for resistance screening in naïve patients is a strategy that should be considered. Cost-effectiveness studies evaluating the different strategies are urgently needed to better inform drug resistance testing recommendations in our setting. Some other issues, related to the epidemiological value of having the protease sequence information, should be considered and modelled in this analysis.

Our study has several limitations. First, we have studied a subset of patients included in CoRIS, as a FASTA sequence was only available for 37.8% of the patients. Restricting to years after 2007, when the recommendation to screen for resistance in all patients was made, minimises the possibility of selective testing of patients with higher probability of resistance. Higher testing in younger males, MSM of Spanish origin and higher education could reflect clinicians' preferences and practices and higher agency of these patients. However, we cannot rule out that selection bias persists at a certain scale, resulting in an infra-estimation of resistance rates. Second, our sequencing data have been obtained through population sequencing and we have not investigated the presence of minor populations. As minor variants are known to have a greater impact on resistance to NNRTIs in naïve patients [24], it is very unlikely that using this technology our main observations could have affected resistance to the other classes, in particular for the PI class. Finally, higher time since HIV infection can limit our ability to find resistance mutations that were present at infection. Unfortunately, we did not have information on the date of infection for most of the patients, although higher CD4 counts, lower percentage of patients in CDC C stage and a slightly higher percentage of recent infections in the latter calendar years could indicate an earlier HIV diagnosis. The fact that we have not found a decreasing trend in TDR (as of WHO list) indicates that this bias, if existing, is probably of small magnitude.

In summary, clinically relevant resistance to ART first line regimens in Spain estimated using the Stanford HIV resistance database algorithm has been found to be lower than the one we expected when looking at the WHO surveillance mutations list, and with a significant trend to a decline through the period 2007–2011. As PI-based first line regimens have shown the highest therapeutic barrier and primary resistance to first-line PIs has been found to be below 1%, we believe that there is a need to question the recommendation of screening for TDR in the protease gene in our setting, and that cost-effectiveness studies need to be carried out to inform evidence-based recommendations.
